# Rev Variation during Persistent Lentivirus Infection

**DOI:** 10.3390/v3010001

**Published:** 2011-01-11

**Authors:** Susan Carpenter, Wei-Chen Chen, Karin S. Dorman

**Affiliations:** 1 Department of Animal Science, Iowa State University, Ames, IA 50011-3260, USA; 2 Department of Statistics, Iowa State University, Ames, IA 50011-1210, USA; E-Mail: snoweye@iastate.edu (W.-C.C.); 3 Department of Genetics, Development, and Cell Biology, Iowa State University, Ames, IA 50011-3260, USA; E-Mail: kdorman@iastate.edu

**Keywords:** lentivirus, Rev, equine infectious anemia virus, selection, immune evasion, overlapping reading frames

## Abstract

The ability of lentiviruses to continually evolve and escape immune control is the central impediment in developing an effective vaccine for HIV-1 and other lentiviruses. Equine infectious anemia virus (EIAV) is considered a useful model for immune control of lentivirus infection. Virus-specific cytotoxic T lymphocytes (CTL) and broadly neutralizing antibody effectively control EIAV replication during inapparent stages of disease, but after years of low-level replication, the virus is still able to produce evasion genotypes that lead to late re-emergence of disease. There is a high rate of genetic variation in the EIAV surface envelope glycoprotein (SU) and in the region of the transmembrane protein (TM) overlapped by the major exon of Rev. This review examines genetic and phenotypic variation in Rev during EIAV disease and a possible role for Rev in immune evasion and virus persistence.

## 1. Introduction

Lentiviruses are characterized by high rates of mutation, recombination, and replication, resulting in multiple, diverse populations of viral variants that rapidly adapt to changes in the host environment. Understanding virus and host factors that shape the evolution and selection of virus variants *in vivo* is an essential component of preventive and therapeutic strategies to control lentivirus infections in humans and animals. Equine infectious anemia virus (EIAV) possesses common features of the *Lentiviridae* subfamily of retroviruses, including a complex genome organization, tropism for cells of the monocyte/macrophage lineage, and establishment of a persistent, life-long infection. Many lentivirus infections are characterized by a slow, chronic disease course, but infection of horses with EIAV can result in an acute, dynamic disease characterized by recurring cycles of viremia, fever, and thrombocytopenia. Most animals eventually gain control of viral replication, progressing to a clinically inapparent stage of disease, yet remain carriers of the virus for life. The dynamics of clinical disease and immune control make EIAV a good model to study the role of both host and viral mechanisms contributing to lentiviral persistence and pathogenesis.

Genetic variation has been observed in the EIAV *rev/tm* overlapping reading frames, which encode the regulatory protein Rev and the cytoplasmic tail of the transmembrane (TM) protein [1–3]. Rev is an essential regulatory protein required for nucleocytoplasmic transport of incompletely spliced viral mRNAs encoding structural proteins. Variation in HIV-1 Rev has been shown to down-regulate the expression of viral late genes and alter sensitivity to Gag-specific cytotoxic T lymphocytes (CTL) [4]. In addition, CTL epitopes have been identified within HIV-1 Rev [5], as well as within EIAV Rev [6]. Genetic changes within *rev* may facilitate immune evasion directly by altering CTL epitopes in Rev, and/or indirectly through altering Rev nuclear export activity and expression of structural proteins. In this review, we focus on the variation and evolution of the *rev* gene over the course of EIAV infection *in vivo*. Our overriding hypothesis is that Rev variation contributes to viral persistence and survival in the host, so that understanding how and why *rev* varies may be key to effective antilentiviral strategies.

## 2. Genetic Variation Alters Rev Activity

Overlapping reading frames are expected to be more conserved than single reading frames as each mutation has an increased risk of causing a deleterious mutation and very few mutations are truly synonymous [7]. When one of the reading frames encodes a protein essential for virus replication, such as Rev, additional stabilizing selection could further reduce variation. Thus, it was surprising to find the second exon of *rev* exhibited high diversity, sometimes comparable to the highly variable surface protein [1,2]. Most of the genetic variation in the *rev/tm* overlapping reading frames occurred outside known functional domains of Rev, and was thought to be of minimal consequence for Rev activity. However, Belshan *et al.* [3] found that polymorphisms in *rev/tm* resulted in significant increases or decreases in Rev activity. This supported the hypothesis that variation in Rev may be an important mechanism for regulation of virus replication *in vivo* and provided a basis for more extensive analyses of Rev variation in experimentally infected horses [8–10].

## 3. Longitudinal Studies of Rev Variation *in Vivo*

### 3.1. Rev Subpopulations Evolve During Disease Progression, Sometimes Coexisting or Recurring

**Figure 1:**
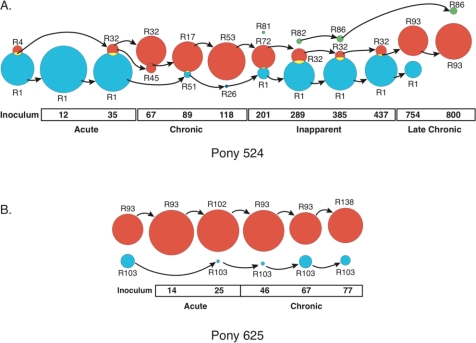
Partition analysis identifies two co-existing sub-populations of *rev* variants [9]. The groups present at each time point were found using the program PAQ [9,12]. The relative size of each group represents the proportion of the population contained within the group at that time point. The central Rev variant for each group is shown, and the arrows show from which group the central variant likely evolved. Groups that overlap indicate that both groups share at least one variant. The different groups are designated Group 1 (red), Group 2 (blue), and Group 3 (green). The day post-infection and clinical stages of infection are indicated. (**A**) Partition analysis of pony 524 *rev* nucleotide variants at sequential times following infection. (**B**) Partition analysis of pony 625 *rev* nucleotide variants at sequential times following infection.

Longitudinal analysis of Rev variation *in vivo* revealed that novel *rev/tm* variants arose throughout infection [8,9]. Overall, genetic variation in Rev was characterized by a low level of synonymous changes in the majority of Rev codons, punctuated by a high rate of non-synonymous changes at a limited number of codons [10]. The marked variation in Rev was not accompanied by co-variation in the Rev-Responsive Element (RRE) [9,11]. Detailed longitudinal analysis in two experimentally infected horses suggested that the *rev/tm* population was comprised of two distinct sub-populations that co-existed during infection [9]. One sub-population, Group A, appeared to accumulate changes in a linear, time-dependent manner, while the other sub-population, Group B, evolved radially from a common variant. Over time, the two sub-populations cycled in predominance coincident with changes in the disease state (Figure 1), suggesting that the two groups differed in selective advantage. When serum from the first pony (pony 524) was used to infect a second (pony 625), the populations were seen to persist through transmission events.

### 3.2. Sub-population Rev Variants Differ in Nuclear Export Activity and Replication Phenotype

**Figure 2:**
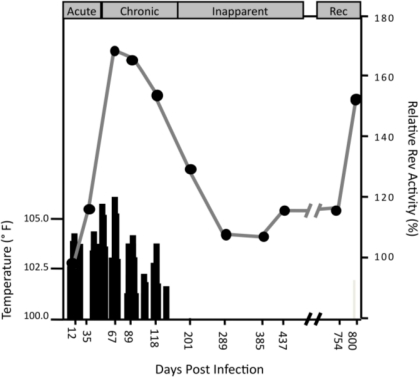
Changes in Rev activity during progression of clinical disease [8]. *In vitro* biological activity of Rev variants from pony 524 was quantified in transient transfection assays using 293T cells as previously described [3]. Briefly, Rev variant cDNAs were co-transfected with a chloramphenicol acetyltransferase (CAT) reporter plasmid (pERREAll) in HEK293T cells and CAT activity was quantified as percent acetylation. The results are presented as a percentage of activity compared to R1, the founder variant in the inoculum. The overall Rev activity for each time point was calculated as the average Rev activity of tested variants, weighted by the frequency of the tested variant in the sampled population. Solid bars indicate the rectal temperature of pony 524. The clinical stages of disease are indicated as: Acute, Chronic, Inapparent, and Rec (recrudescent).

Biological characterization of Rev variants representative of Group A and Group B were undertaken to determine if the sub-populations differed in Rev phenotype. Seventeen variants from the two ponies, representing 54–67% of the sampled *in vivo* population, were tested for Rev activity in transient expression assays. For 16 of the 17 variants tested, Rev activity segregated according to the genetic groups identified by phylogenetic and partition analyses. Further, Rev activity of all variants from Group A was significantly higher than the activity of all variants from Group B (p < 0.0001). To examine possible changes in Rev activity during disease progression, the activity of each tested variant and its frequency in the population was used to calculate an average Rev activity at each of the sampled time points [8]. Comparison of the average Rev activity with clinical parameters of disease progression indicated that Rev activity was highest during the recurrent febrile and late febrile stages of EIA (Figure 2). Rev activity was correlated with temperature (p = 0.007), and Rev activity in the recurrent febrile stage was significantly higher than Rev activity in the inapparent stage of infection (p = 0.010). Additionally, replication competent chimeric viruses containing *rev/tm* variants representative of Group A replicated faster, and to higher levels, than Group B viruses *in vitro*. The co-existence of genetically distinct viral sub-populations that differ in phenotype provides great adaptability to environmental changes within the infected host.

## 4. Selection in an Overlapping Reading Frame

Given the high diversity in the second *rev* exon and the strong association between Rev phenotype and clinical disease, we wanted to determine whether the diversity was caused by selection for change in *rev*, *tm*, or both. Standard methods for detecting selection that test for an elevated rate of nonsynonymous relative to synonymous mutations—the so-called dN/dS ratio [13]—cannot work in overlapping reading frames where most synonymous mutations in one frame are nonsynonymous in the other. Failure to account for cryptic selection on synonymous mutations can lead to false evidence for selection at the protein level [14–16]. Indeed, purifying selection on HIV-1 RNA functional elements contained within genes are correlated with unusually large dN/dS ratios [17].

### 4.1. Model for Estimating Selection in Overlapping Reading Frames

Hein [7] extended the Li counting method [18] and Pedersen [19] extended the likelihood-based methods [20,21] for estimating selection in open reading frames to handle overlapping frames. Both models classify mutations into four categories: SS mutations are synonymous in both reading frames, SN are synonymous in the first reading frame, nonsynonymous in the second, NS the opposite, and NN are nonsynonymous in both. Since the Pedersen model [19] only works on pairs of sequences, Sabath [22] approximated the Peterson model in order to analyze more data at the cost of assuming independent evolution in each overlapping reading frame. We have recently proposed an alternative approximation to the Pedersen model that assumes some sites are invariant (cannot mutate) and all mutations are observed (a form of parsimony) [23]. While these approximations are not reasonable for highly variable genes, they represent a reasonable compromise in relatively conserved overlapping regions.

Briefly, the model takes an alignment of wholly overlapped reading frames. One sequence is identified as the ancestral sequence; the consensus sequence will often suffice if none is known. The alignment is then partitioned at non-segregating sites into a collection of independently evolving variable-length fragments. The non-segregating sites used to fragment the alignment are treated as invariant sites that can never mutate. There are multiple choices of invariant sites, but the results are minimally affected by the choice (data not shown). Under the parsimony assumption, we have derived the probability of transitioning between any pair of fragments for a model that has specific rates for transversions relative to transitions and NS, SN, and NN relative to SS mutations. For example, one may interpret the relative rate of NS to SS mutations as a dNS/dSS ratio. All sequences are assumed to evolve independently from the ancestral sequence. This approximation avoids the need to infer a complex phylogenetic tree or network, but may tend to overweight ancient relative to recent mutations.

### 4.2. Evidence for Selection in Both Rev and TM

**Figure 3:**
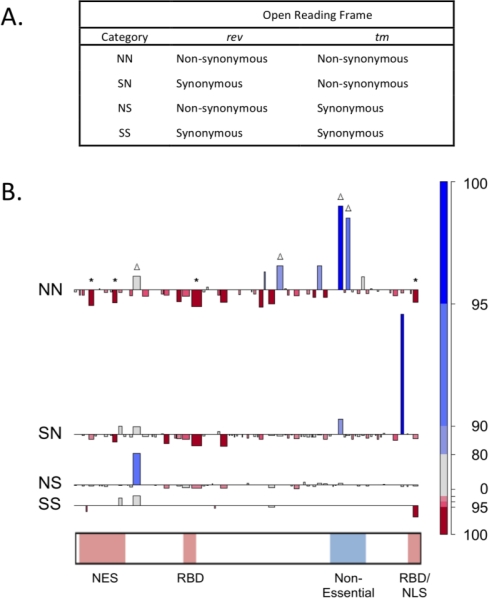
Analysis of mutations in *rev/tm* overlapping reading frames. (**A**) Categories of mutations in overlapping reading frames indicating the type of mutation in Rev and TM. (**B**) The difference between the observed and expected number of each mutation type (SS, NS, SN, and NN) is plotted for each of the fragments along the Rev open reading frame. Expected numbers were computed as the mean of 160 simulations of the model using the maximum likelihood estimated parameters. Observed numbers were counted directly from the Pony 524 *rev/tm* alignment. The blue bars above the line indicate more mutations of the designated type were observed than expected in that fragment whereas red bars below indicate fewer mutations were observed than expected. Width of the block indicates the length of the fragment. All differences were ranked from smallest to largest. Dark blue indicates the top 5% of differences; dark red indicates bottom 5% of differences. Functional domains important for Rev activity are depicted in red and the non-essential region is blue. Asterisks indicate fragments containing amino acids shown to be essential for Rev activity, while delta symbols indicate amino acids shown to modulate Rev activity.

Analyses of all 146 *rev/tm* sequences obtained during EIAV infection of pony 524 support the full model with one transversion/transition bias and three distinct selection ratios: dNS/dSS, dSN/dSS, and dNN/dSS, where the first letter denotes changes in *rev*, and the second letter denotes changes in *tm* (Figure 3). In particular, the hypothesis of independent selection in the two reading frames is rejected. Interestingly, there appears to be greater diversifying selection on the second frame encoding *tm*, since the relative rate of SN (0.26) mutations is higher than NS mutations (0.13), however there is a surprisingly high selection or tolerance for doubly nonsynonymous NN mutations (0.47). Indeed, the propensity to NN mutations is the reason for rejecting the hypothesis of independent selection and suggests the virus may have adopted sites that can change both proteins in order to allow evolution in these genes. It is remains unclear which of the two protein products is the selective target of these NN changes.

By comparing the observed number of each mutation to the expected under the full, fitted model, it is possible to identify fragments with unusually low or high numbers of each type of mutation (Figure 3). One fragment near the end of the overlap with more SN mutations than expected could indicate selection for change at this site in TM. Another fragment just downstream of the Rev nuclear export signal (NES) has elevated NS mutations, indicative of possible selection in Rev. Four fragments (marked with Δ in Figure 3) with more NN mutations than expected, including two fragments in the nonessential region of Rev [24], were found to significantly change Rev activity in transient expression assays [10]. Several fragments have fewer mutations than expected, possibly indicating essential regions. Four fragments (marked with asterisks in Figure 3) deficient in NN mutations align with known lethal mutations in the NES and RNA binding domains (RBD) of Rev.

## 5. Genetic Determinants of Rev Phenotype

The results of the selection analyses suggested fragments that may be under selection in the Rev protein. Genetic variation was observed in 121 of 135 Rev codons; however, the mutations at these positions occurred at a low frequency (less than 2%), and many of these were synonymous changes; 70 amino acid positions were 100% conserved. However, there were nine highly variable amino acid positions in Rev sequences extracted from pony 524, which (together) experienced a total of ten distinct high frequency amino acid mutations from the inoculum consensus [10]. All but one of the highly variable amino acid positions observed *in vivo* were found outside the known functional domains of EIAV Rev, and four of the highly variable positions occurred within a non-essential region of Rev [24–27]. Nonetheless, nine of the ten naturally occurring amino acid substitutions were found to significantly alter Rev activity, either as single mutations or in the context of cumulatively fixed mutations [10].

The effect of variation at each of the highly variable positions was assessed in transient expression assays [10]. Seven of the ten amino acid mutations significantly altered Rev phenotype; six increased Rev activity and one decreased Rev activity. All of these changes occurred in fragments found to have somewhat more NN mutations than expected (Figure 3), consistent with selection for effects on Rev activity. The phenotype analyses demonstrated that the majority of Rev mutations observed at a high frequency *in vivo* were alone sufficient to cause significant changes in nuclear export activity. Moreover, EIAV Rev nuclear export activity was found to be highly sensitive to point mutations outside essential functional domains. The presence of multiple mutational pathways to high Rev activity could confer flexibility on a protein whose evolution is constrained by an overlapping reading frame and/or immune epitopes. Further, the complexity of these pathways suggests it will be difficult to predict Rev phenotype based on sequence alone; consequently, the biological significance of Rev variation may be underestimated.

## 6. Rev Variation and Immune Evasion

Although longitudinal studies in a limited number of horses suggest that Rev phenotype contributes to variant selection *in vivo*, the specific selective pressures on Rev are unknown. Genetic changes within *rev/tm* may facilitate immune evasion by directly altering CTL epitopes, and it is likely that some of the observed variation in Rev is due to antigenic escape from Rev-specific CTL. CTL epitopes have been identified within EIAV Rev [6], and a high-avidity CTL response to epitopes within Rev has been associated with low viral loads and mild clinical disease [6]. In one horse, two CTL epitopes were identified and mapped to conserved regions important for Rev activity, and there was little variation observed within the CTL epitopes. The presence of high-avidity CTLs to conserved epitopes may constrain virus options for escape, leading to effective immune control and lack of disease progression. It is important to note that Mealey [6] also observed some variation at epitopes outside known functional domains, including the variable, non-essential region; however, it is not known if these changes alter Rev activity.

A second mechanism by which Rev can contribute to immune evasion is down regulation of structural gene expression. Bobbitt *et al.* [4] demonstrated that variation in HIV-1 Rev activity could alter sensitivity to CTL killing by down regulating viral late gene expression. A single amino acid substitution in HIV-1 Rev resulted in a 2–3 fold reduction in Gag expression and a significant decrease in CTL killing. Further, primary HIV-1 isolates from asymptomatic patients showed reduced Rev activity, reduced levels of Gag expression, and increased resistance to CTL killing. Rev-attenuated phenotypes have been associated with nonprogressive HIV-1 infection [28–30], suggesting that variation in Rev could alter virus replication levels *in vivo* and modify the clinical outcome of infection. The detailed analyses of Rev variation in EIAV infected horses provide basis for further evaluation of mechanisms by which Rev variation may alter levels of virus replication *in vivo* and may contribute to changes in clinical disease. Ongoing and additional studies in EIAV-infected horses will increase our understanding of the role of Rev variation in immune evasion and lentiviral persistence.

## 7. Summary and Conclusions

A number of early studies of EIAV variation *in vivo* [1,2] reported considerable genetic variation in Rev, which is striking considering that the second exon of Rev overlaps with the reading frame encoding the envelope transmembrane protein. Subsequent studies have shown that the genetic variation leads to detectable changes in Rev activity and virus replication. A novel analysis of selection on the overlapping reading frames has revealed a somewhat higher rate of nonsynonymous mutation in *tm*; however, most evolution is occurring through double nonsynonymous (NN) mutations. While the statistical analysis cannot reveal whether the NN mutations are tolerated or selected in one or the other, additional phenotypic analyses have shown the most common NN mutations alter Rev nuclear export activity. In addition, there are multiple configurations of the protein that result in the same Rev activity, which can allow the overlapping reading frames to accommodate other selection pressures, for example, at CTL epitopes or in TM. The detailed analyses of Rev variation in EIAV-infected pony 524 has raised a number of intriguing hypotheses about EIAV evolution and adaption during disease progression in a horse that passed through clearly demarcated stages of disease. Study of additional horses will be needed to confirm these hypotheses or further enrich our understanding of pathogen-host interactions in EIAV and other related lentivirus infections.
